# Evaluation of Root Angulations Through Panoramic Films Using Artificial Intelligence

**DOI:** 10.3390/diagnostics16040634

**Published:** 2026-02-22

**Authors:** Deniz Şevik, Nurullah Akkaya, Ulas Oz, Beste Kamiloglu

**Affiliations:** 1 Department of Orthodontics, Faculty of Dentistry, Near East University, 99138 Mersin, Turkey; 2 Department of Computer Engineering, Applied Artificial Intelligence Research Centre, Near East University, 99138 Mersin, Turkey; 3 Department of Orthodontics, Faculty of Dentistry, Final International University, 99138 Mersin, Turkey

**Keywords:** artificial intelligence, deep learning, panoramic radiography, root angulation, root parallelism, orthodontics, tooth segmentation, convolutional neural networks, automated image analysis

## Abstract

**Background/Objectives**: Accurate evaluation of root angulation is essential for assessing root parallelism and orthodontic treatment outcomes. In routine clinical practice, this assessment is often performed by visual inspection of panoramic radiographs, which is subjective and prone to observer variability. The objective of this study was to develop and validate an artificial intelligence (AI)–based algorithm for automated, quantitative assessment of mesiodistal root angulations on panoramic radiographs and to evaluate its accuracy relative to conventional manual measurements. **Methods**: A total of 214 panoramic radiographs (orthopantomograms), comprising 4280 posterior teeth, were retrospectively selected after applying strict inclusion and exclusion criteria. Individual teeth were automatically segmented using a U^2^-Net–based deep learning architecture. Tooth long-axis orientation was calculated using principal component analysis, with exclusion of the apical third to minimize the influence of root curvature. Angular deviation was measured relative to fixed horizontal reference lines. Manual measurements performed by experienced examiners using 3D Slicer software served as the reference standard. Intra- and inter-examiner reliability, agreement between AI-based and manual measurements, intraclass correlation coefficients (ICC), and Bland–Altman analyses were calculated. **Results**: Manual measurements demonstrated excellent reliability, with intra-examiner and inter-examiner ICC values of 0.972 and 0.963, respectively. Agreement between the AI-based algorithm and manual measurements was also excellent (ICC = 0.941). Bland–Altman analysis showed a mean difference of −0.10°, with 95% limits of agreement ranging from −1.60° to 1.41°, indicating minimal bias and no proportional error. **Conclusions**: The proposed AI-based algorithm provides accurate, objective, and reproducible measurements of posterior tooth root angulations on panoramic radiographs. This approach may support clinical decision-making, reduce observer-related variability, and facilitate efficient assessment of root parallelism in orthodontic practice.

## 1. Introduction

Artificial intelligence (AI) can be defined as the capability of machines to perform complex tasks, including problem-solving, planning, and decision-making, by mimicking human cognitive functions [1,2,3].

Machine learning (ML), a subset of AI, has recently gained popularity in the medical field because of its ability to learn from experience without specific programming [1,4]. Unlike knowledge-based expert systems that rely on predefined rules, ML focuses on improving performance through training data [5].

Deep learning (DL) is an advanced subset of ML algorithms that employs multiple hidden layers of artificial neurons to create a processing network [2]. At its core, DL techniques rely on neural networks, which enable knowledge acquisition through the analysis of data patterns [1]. DL enhances traditional artificial neural networks (ANNs) by incorporating deeper architectures and higher levels of representation, thereby improving data classification and detection performance [4]. In computer vision, convolutional neural networks (CNNs) are particularly effective for image recognition [1], especially with high-resolution images [5].

These developments have led to increased interest in the application of DL models in dental imaging. Consequently, the integration of CNN image processing capabilities into dental radiographs has enabled numerous applications of AI in dentistry and accelerated the image analysis process. The majority of ML algorithms are developed using two-dimensional diagnostic images, including cephalometric, periapical, and orthopantomograms (OPGs).

The assessment of mesiodistal root angulation on OPGs is challenged by inherent limitations, including variable image quality, geometric distortion, and overlapping anatomical structures. Landmark-based and conventional image-processing approaches rely on accurate localization of predefined anatomical points; however, blurred boundaries, apical morphological variations, restorations, and tooth superimposition frequently limit their reliability on OPGs. Because small localization errors may translate into clinically relevant angular deviations, an analysis framework that is less sensitive to local uncertainty is required. Accordingly, a CNN-based semantic segmentation strategy was adopted to enable morphology-driven estimation of tooth long axes rather than reliance on isolated landmarks. In this context, the U^2^-Net architecture was selected due to its nested multi-scale feature extraction design, which supports stable segmentation of structures with complex contours and heterogeneous boundaries under variable imaging conditions, making it well suited for root angulation assessment on OPGs [6].

OPGs are generally considered a principal diagnostic tool [1], as they provide extensive diagnostic information covering a wide area of dentomaxillomandibular structures and surrounding tissues on a single image [1,7]. They also offer advantages such as a relatively low radiation dose, short imaging time, and minimal burden to the patient [4].

Surveys indicate that 57.9% of orthodontists take progress OPGs, while 79.1% obtain post-treatment OPGs to evaluate the maxillary and mandibular dentition [8]. One contributing factor to this widespread usage may be that, apart from periapical radiographs or three-dimensional imaging, OPGs are considered the most practical method for assessing root parallelism [9].

In the literature, tooth angulation has received increased attention, especially after Andrews included crown angulation as one of the Six Keys of Normal Occlusion. Although root position was not directly considered in this classification [10], root parallelism was later accepted as a direct criterion for evaluating orthodontic treatment outcomes [9].

Ensuring root parallelism contributes to the correct alignment of the teeth within their apical bases, thereby supporting a functionally stable and esthetically pleasing occlusion [11,12]. Tight interproximal contacts, even distribution of occlusal stresses, and improved long-term stability have all been associated with roots that are parallel to adjacent teeth [12,13,14].

Apical convergence or occlusal divergence may complicate the maintenance of tight interproximal contact points and adequate oral hygiene, potentially leading to inflammatory periodontal diseases [13,15]. On the other hand, apical divergence or occlusal convergence can result in open embrasures and black triangles, promote food retention, and negatively affect periodontal health [16].

In routine clinical practice, root angulations on OPGs are often assessed by rapid visual inspection in the absence of protractor-based or computer-aided measurement tools. The aim of this study was to develop and validate an AI-based automated algorithm for the assessment of mesiodistal root angulation on OPGs and to evaluate its accuracy relative to conventional manual measurements. By objectively identifying teeth that disrupt root parallelism and quantifying the degree of angular correction required, the proposed approach seeks to support clinical decision-making, enhance treatment quality and long-term stability, minimize observer-dependent errors, and enable a faster and more standardized evaluation process.

## 2. Materials and Methods

A request was filed to and approved by the Near East University Scientific Research Evaluation Ethics Committee in compliance with the 1964 Helsinki Declaration on medical research ethics (decision number and date YDU/2025/133-1927).

### 2.1. Preparation of Dataset

A total of 7.751 OPGs were collected from the Orthodontic Department Archive at Near East University. To minimize potential device-related bias, the OPGs were obtained using 3 different imaging devices. The collected data consisted of patients with natural permanent dentition. Patients meeting any of the following criteria were excluded:

1. Primary or mixed dentition.
2. Retained primary teeth.
3. Presence of impacted teeth (except third molars).
4. Multiple missing teeth.
5. Severe caries preventing determination of the tooth’s long axis.
6. Pathologic lesions affecting root position.
7. Dental anomalies affecting tooth number (e.g., hypodontia, supernumerary teeth) or morphology (e.g., mesiodens, fusion).
8. Syndromes affecting dentition (e.g., cleft lip and palate).
9. Presence of imaging artifacts.

In total, 214 OPGs met the inclusion criteria and were included in the study. All images were pseudonymized prior to the measurement process.

### 2.2. Model Pipeline

The input OPGs were preprocessed by resampling them to a fixed resolution of 512 × 1024 pixels and normalizing them to single-channel grayscale intensity values. Semantic segmentation of individual teeth was performed using a U^2^-Net encoder–decoder architecture with nested residual U-blocks and deep supervision at intermediate decoder stages. The network was trained end-to-end using the AdamW optimizer (learning rate = 2 × 10^−4^) with Dice loss as the objective function over 250 epochs, employing an 80/10/10 training-validation-test split. The output consisted of a 33-class dense prediction map corresponding to 32 tooth instances following FDI notation, along with the background class.

**Figure 1:**
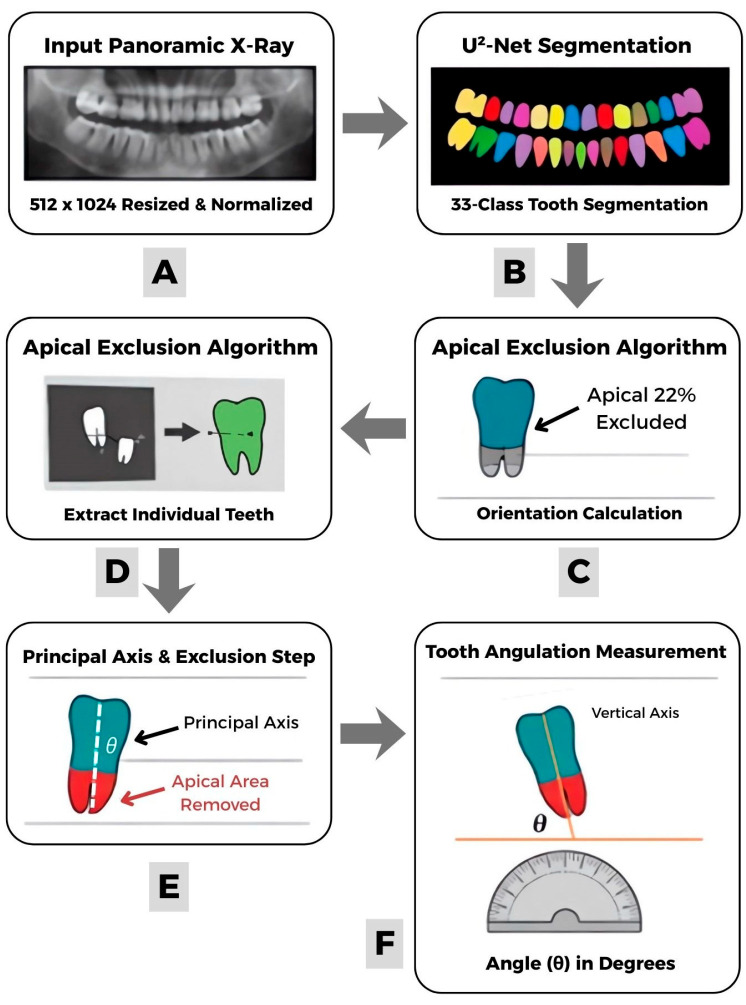
Model pipeline for automated tooth angulation measurement. (**A**) Input panoramic radiograph resized and normalized to a fixed resolution. (**B**) Tooth segmentation using a U^2^-Net architecture, producing a 33-class dense prediction map. (**C**) Apical exclusion algorithm applied to remove the apical portion of each tooth mask prior to orientation analysis. (**D**) Connected component analysis used to extract individual tooth instances. (**E**) Principal axis calculation performed after apical exclusion to define the tooth’s long axis. (**F**) Final tooth angulation (θ) measured relative to the selected reference line.

Following segmentation, individual tooth instances were extracted using connected component analysis on each class-specific binary mask. An orientation-aware apical exclusion algorithm was developed to estimate the long-axis orientation while reducing the influence of root morphology. For each segmented tooth region, we computed the principal axis orientation (θ) using second-order central moments derived from region properties. Pixel coordinates were projected onto this principal axis, and the apical 22% of the tooth extent along this axis was excluded based on anatomical position—pixels at the minimum projection values for maxillary teeth and maximum projection values for mandibular teeth. Tooth angulation was subsequently quantified as the angular deviation of the processed region’s principal axis from the vertical image axis, expressed in degrees. This pipeline (Figure 1) enables automated, reproducible measurement of tooth inclination without manual landmark annotation.

### 2.3. Method of Manual Measurement

To measure root angulations, it is first necessary to determine the long axes of the teeth and to define a reference line. When establishing the long axes, each tooth was delineated by connecting the midpoint of the mesiodistal diameter of the dental crown along the root canal path or bifurcation. The apical third of the roots was excluded because this region frequently exhibits curvature and dilacerations. Inclusion of the apical portion may introduce multiple possible axial orientations for the same tooth, thereby reducing the accuracy and reproducibility of angulation measurements. Therefore, only the cervical and middle thirds of the roots were used to define the long axis, as recommended in previous orthodontic studies assessing root parallelism [12,17].

Two horizontal fixed borders of the OPG were selected as reference lines, as they are consistently reproducible across all images and independent of patient head orientation. Accordingly, the upper border of the OPG, parallel to the midfilm horizontal X-line, was used as the reference line for the maxilla, whereas the lower border of the OPG served as the reference line for the mandible.

**Figure 2:**
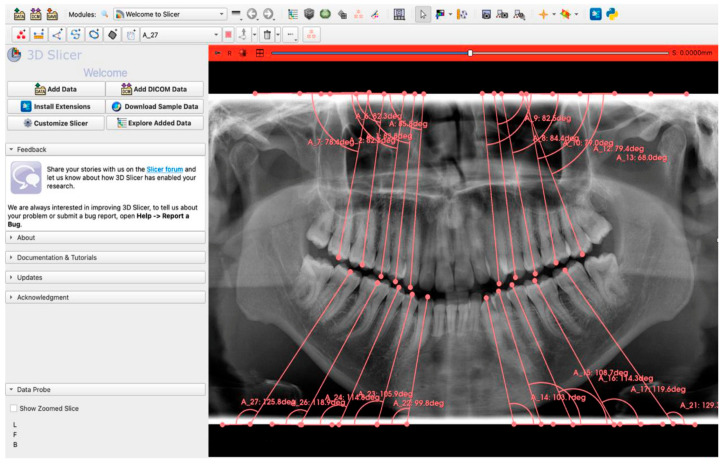
Manual measurement of mesiodistal root angulations on OPGs using 3D Slicer software (version 5.8.1).

For each OPG, angular measurements were performed on the canines, first and second premolars, and first and second molars in both the maxillary and mandibular arches (Figure 2).

A previously trained AI-based system was employed to automatically segment each tooth, determine its long axis, and measure axial inclination relative to the selected reference line. Segmentation performance was evaluated using the Dice similarity coefficient (DSC), a widely accepted overlap-based metric for assessing spatial agreement in medical image segmentation tasks. The Dice coefficient was selected as the primary metric because it directly quantifies the overlap between predicted tooth masks and reference annotations and is particularly relevant for morphology-based analyses such as long-axis estimation. The present study employed a previously validated tooth segmentation framework without architectural modification.

To evaluate the robustness of the fixed-border reference line approach to variations in patient head positioning and panoramic projection geometry, a sensitivity analysis was performed using repeat OPGs from the same individuals. For patients with two available OPGs acquired at different time points, AI-derived mesiodistal root angulation measurements were calculated for each image and compared on a tooth-by-tooth basis. Absolute differences between repeated measurements were summarized across all teeth.

**Figure 3:**
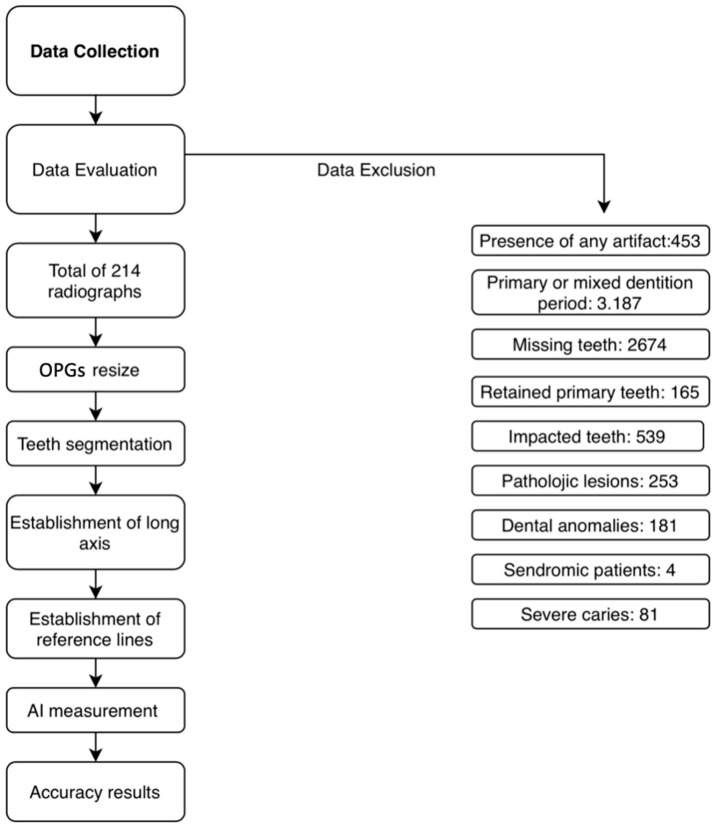
Overview of data processing and analysis steps.

The same measurements were independently performed manually by an experienced examiner (D.Ş.) using 3D Slicer software (version 5.8.1), an open-source medical image analysis platform; these manual measurements served as the reference standard for accuracy assessment. To evaluate inter-rater reliability, a second examiner (B.K.) independently measured 20% of the dataset. The entire dataset was then processed by the AI algorithm, and the resulting measurements were quantitatively compared with the reference standard values to assess AI accuracy. The entire workflow of the study is summarized in Figure 3.

### 2.4. Statistical Analysis

All statistical analyses were performed using SPSS software (version 21.0; IBM Corp., Armonk, NY, USA). Continuous variables were expressed as mean ± standard deviation, and 95% confidence intervals (CIs) were calculated where appropriate.

The reliability of manual measurements was assessed by evaluating both intra-examiner and inter-examiner agreement. Intra-examiner reliability was determined by remeasuring 20% of the dataset after a four-week interval by the same examiner (D.Ş.), while inter-examiner reliability was assessed by a second independent examiner (B.K.), who evaluated the same subset of data. Reliability analysis was performed using intraclass correlation coefficients (ICCs) with the following settings: absolute agreement and single measures.

Agreement between the AI-based algorithm and manual reference measurements was evaluated using ICC with absolute agreement. Given that the AI algorithm represents a fixed measurement method, a two-way mixed-effects model was applied for the AI–manual agreement analysis (ICC [3,1]). ICC values were interpreted as follows: <0.50, poor; 0.50–0.75, moderate; 0.75–0.90, good; and >0.90, excellent agreement.

To further assess agreement and identify potential systematic bias between AI-based and manual measurements, Bland–Altman analysis was performed. The mean difference (bias) and the 95% limits of agreement (mean difference ± 1.96 standard deviations) were calculated and graphically illustrated.

## 3. Results

After application of the exclusion criteria (Table 1), a total of 214 OPGs comprising 4280 teeth were included in the final analysis. Measurements of mesiodistal root angulation were successfully obtained for all included teeth using both the AI-based algorithm and manual assessment.

### 3.1. Tooth Segmentation Performance

The tooth segmentation performance of the AI model demonstrated high accuracy (DSC = 0.95), derived from a previously validated implementation of the same segmentation algorithm reported in reference [3], rather than recalculated on the present dataset. This result indicates strong spatial agreement between the predicted tooth masks and the reference annotations, supporting the reliability of the subsequent morphology-based mesiodistal root angulation measurements.

Given the strong mathematical correspondence between the Dice coefficient and the Jaccard index (IoU), additional overlap-based metrics were not separately reported. Precision and recall metrics were also omitted, as segmentation was performed at the tooth-instance level and overlap accuracy relevant to long-axis estimation was sufficiently captured by the Dice metric. Visual inspection of the segmentation outputs did not reveal systematic failure patterns that would compromise angulation analysis.

### 3.2. Sensitivity Analysis of Reference Line Robustness to Acquisition Variability

In the repeat OPG sensitivity analysis (*n* = 100 teeth from 5 patients), the mean absolute difference in AI-derived angulation measurements between two acquisitions of the same individuals was 3.16°, with a 95th percentile of 4.85°. The maximum observed deviation was 6.72°. Overall, 66% of tooth-level measurements showed absolute differences greater than 1°, and 53% exceeded 2°, illustrating the magnitude of variability attributable to differences in head positioning and panoramic projection geometry. intra-examiner and inter-examiner reliability analyses of manual measurements demonstrated excellent agreement.

### 3.3. Reliability of Manual Measurements

Intra-examiner reliability analyses of manual measurements demonstrated excellent agreement. The intra-examiner ICC was 0.972, and the inter-examiner ICC was 0.963.

### 3.4. Agreement Between AI-Based and Manual Measurements

Agreement between the AI-based algorithm and manual measurements was excellent, with an ICC of 0.941.

**Figure 4:**
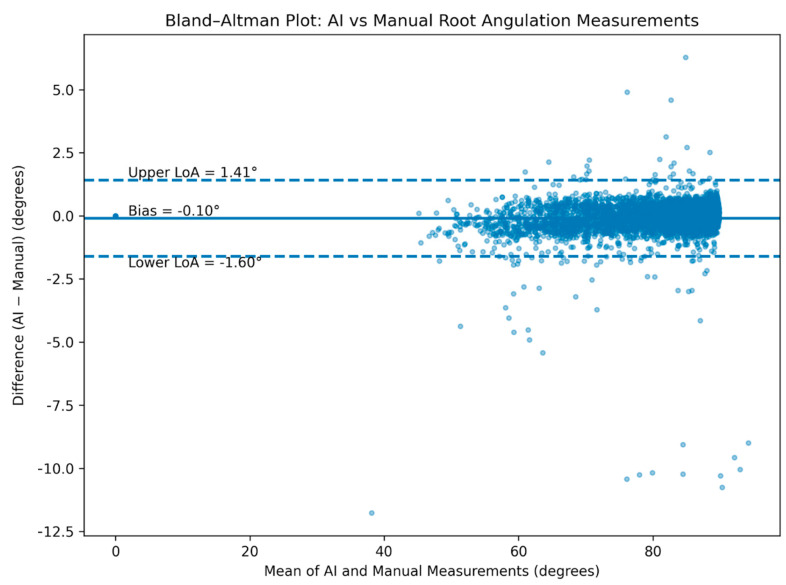
The Bland–Altman plot.

The Bland–Altman analysis revealed a mean difference (bias) of −0.10°, with 95% limits of agreement ranging from −1.60° to 1.41°. The Bland–Altman plot (Figure 4) showed that the vast majority of differences lay within the limits of agreement, with no evidence of proportional bias across the range of measured angulations.

### 3.5. Tooth-Type–Specific Agreement Analysis

To further explore clinically relevant performance patterns beyond global agreement metrics, tooth-type–specific analyses were performed. Measurements were grouped according to tooth type: canines, first premolars, second premolars, first molars, and second molars.

Across all tooth groups, excellent agreement between AI-based and manual measurements was observed. The mean absolute error was 0.39° for canines, 0.36° for first premolars, 0.39° for second premolars, 0.46° for first molars, and 0.53° for second molars. Corresponding ICC values ranged from 0.921 to 0.958, indicating excellent reliability across all tooth types, with slightly higher error magnitudes observed in the molar regions.

### 3.6. Arch-Wise and Side-Wise Agreement Analysis

Arch-wise comparisons demonstrated similarly high agreement for both maxillary and mandibular teeth. The mean absolute error was 0.40° for maxillary teeth and 0.45° for mandibular teeth, with ICC values of 0.993 and 0.997, respectively.

Side-wise analyses revealed no meaningful asymmetry between the left and right sides. The mean absolute error was 0.43° for left-sided teeth and 0.41° for right-sided teeth, with ICC values of 0.996 and 0.997, respectively, indicating consistent performance of the AI-based system across both sides of the dental arches.

## 4. Discussion

In routine orthodontic practice, root parallelism is primarily evaluated through rapid visual assessment of OPGs [9]. Since angle measurement tools are often unavailable, this approach increases the potential for observer-dependent variability in clinical assessment.

AI applications in orthodontics have mainly focused on cephalometric analyses, automated landmark detection, and treatment planning [18], while AI-based quantitative assessment of root angulations remains limited.

This study addresses an existing gap by introducing a clinically applicable, AI-based method for the quantitative evaluation of root angulation across the permanent dentition using routinely acquired OPGs.

Although periapical radiographs and CBCT provide superior image detail, their routine use in orthodontic records is limited due to higher radiation exposure, cost, and limited availability [15]. Despite inherent geometric distortions and magnification artifacts that may affect accuracy [19], OPGs are still regarded as the most practical means for this assessment in routine orthodontic practice [9], due to their lower radiation dose, lower cost, and widespread availability for evaluating root angulations before, during, and after treatment [20].

A review of the existing literature reveals that a previous study reported an AI-based system for automated molar angulation measurement on OPGs, particularly for third molar eruption prediction [21]. However, to date, there is limited evidence on AI-based systems specifically designed to quantify root angulations across the permanent dentition and to validate their performance against experienced orthodontic observers in the context of post-orthodontic root parallelism assessment.

To the best of our knowledge, only one previous study has applied AI to evaluate root angulation; however, that approach relied on categorical classification (mesial, distal, or straight) rather than quantitative angle measurement. In contrast, the present study provides numerical angulation values by determining the long axis of each tooth and measuring its angle relative to a fixed horizontal reference plane on OPGs. This quantitative, clinically oriented approach is intended to support objective evaluation of root parallelism, offering guidance not only for the finishing phase of orthodontic treatment but also for broader clinical applications such as prosthetic planning and implant placement [22].

In previous methodological approaches, such as that described by Tao et al. [11], root parallelism was assessed using anatomical reference structures and the entire long axis of the teeth. By comparison, in the present study, the apical third was intentionally excluded to minimize the potential influence of root dilacerations on measurement accuracy. To reduce potential sources of error associated with head positioning and limited landmark visibility on OPGs [20,23], fixed reference lines parallel to the upper and lower horizontal borders of the image were employed. As inter-root angulations do not correspond to established anatomical normative values, the use of non-anatomical reference lines should be regarded not as a limitation but as a methodological approach aimed at improving measurement accuracy and reproducibility. Moreover, the accuracy with which such anatomical landmarks are detected by AI systems represents an additional source of uncertainty that may influence absolute angulation values. Since the primary objective of the present study was not to determine absolute angulation values but to assess the relative consistency and agreement of root angulation measurements, a stable image-based reference line was preferred.

In line with this methodological choice, the repeat-panorama sensitivity analysis provided empirical support for the use of a fixed image-based reference line. The observed differences in AI-derived angulation measurements between repeated acquisitions of the same individuals primarily reflected variability related to patient head positioning and panoramic projection geometry, rather than instability of the proposed algorithm.

These findings underscore the inherent sensitivity of panoramic-based angular measurements to real-world acquisition conditions and support the rationale for prioritizing measurement consistency and reproducibility over absolute angulation values. Accordingly, the fixed-border reference line approach should be interpreted as a strategy to mitigate acquisition-related variability within the known limitations of OPGs.

In the previously reported AI-based approach, root angulation was calculated using vectors derived from crown and root tip keypoints, with an additional vector constructed relative to an occlusal line reference. However, keypoint-based measurements may not always accurately represent the true long axis of the tooth. This limitation may be particularly evident in teeth with multiple roots, where consistent identification of a single root apex can be challenging. In addition, the crown tip does not necessarily align with the tooth’s long-axis orientation, which may introduce variability in angulation measurements on panoramic radiographs [23].

While the previously reported AI-based study included all tooth groups in its analysis [23], the present study focused specifically on posterior tooth groups. This decision was based on two main reasons. First, in orthodontic practice, clinically relevant root convergence or divergence is most commonly observed in extraction cases, where posterior teeth often fail to achieve ideal root parallelism [11,15]. Therefore, the posterior dentition was specifically targeted. Second, the anterior region, particularly the incisor area, is more susceptible to distortion, superimposition, and reduced structural clarity on panoramic radiographs, which complicates consistent identification of the root apex and may adversely affect the accuracy of angulation measurements.

Previous AI-based studies evaluating tooth angulation on panoramic radiographs have used fully convolutional neural networks with pretrained backbones, such as ResNet-101 for molar angulation analysis and Keypoint R-CNN for crown and root landmark detection. In the present study, a U^2^-Net encoder–decoder architecture, which is known for its proven performance in background removal in medical image segmentation, was employed for semantic segmentation of individual teeth.

In this study, the excellent intra-examiner and inter-examiner reliability of manual measurements, along with the high level of agreement between the AI-based algorithm and manual measurements (ICC = 0.941), support the reliability of the reference measurements and suggest minimal systematic differences between the two approaches. These findings may be attributed to the standardized and reproducible measurement protocol, the use of direct angular measurements expressed in degrees rather than categorical classification, and the exclusion of radiographs with features likely to compromise measurement accuracy.

One limitation of the present study is the relatively small sample size, which may be attributed to the application of broad and strict exclusion criteria. These criteria were intentionally defined to ensure adequate image quality, anatomical integrity, and reliable determination of tooth long axes, all of which are essential for accurate root angulation assessment and AI validation. Although this approach reduced the number of eligible OPGs, it resulted in a more homogeneous dataset and enhanced measurement reliability.

As the analysis was limited to posterior tooth groups, the applicability of the findings may be restricted in clinical contexts that require detailed anterior root angulation assessment.

Another limitation of the present study is that, although data were obtained from multiple OPG devices, the performance of the proposed model may not be directly generalizable to all panoramic imaging systems used in clinical practice.

Given the limited number of AI-based studies dedicated to quantitative root angulation analysis, opportunities for direct comparison were restricted. In particular, the most methodologically similar study focused on categorical classification of root orientation rather than quantitative angulation measurement, thereby limiting comprehensive comparative evaluation.

Future research may expand the proposed approach to larger datasets, different imaging systems, and anterior teeth, as well as incorporate three-dimensional imaging to evaluate buccolingual root inclinations. In addition, longitudinal studies may further clarify the role of AI-based root angulation assessment in orthodontic treatment monitoring and outcome evaluation.

The exclusion criteria applied in this study, including severe caries preventing reliable determination of the tooth long axis and the presence of multiple missing teeth, represent clinical conditions that may complicate long-axis identification on OPGs and introduce additional variability into angular measurements. As the relative contribution of these factors to overall measurement uncertainty has not yet been clearly established, the present study deliberately focused on high-quality OPGs free from such confounding variables in order to first evaluate the baseline measurement performance of the proposed AI-based root angulation framework under controlled conditions.

While this approach enhances internal validity and measurement reliability, it may limit external validity in routine clinical settings, where restorations, implants, image artifacts, developmental anomalies, and compromised image quality are frequently encountered. Accordingly, the findings of this study should be interpreted as reflecting optimal performance conditions. Future investigations incorporating more heterogeneous and clinically representative datasets, as well as retraining or fine-tuning of the segmentation framework, will be necessary to improve robustness and extend the clinical applicability of the proposed AI-based approach.

Clinically, the proposed system can be integrated into routine OPG analysis without the need for specialized hardware, enabling its use on standard clinical computers. The algorithm may support pre-treatment assessment of root positions and mesiodistal angulations, as well as mid-treatment decision-making in cases requiring angulation correction through bracket repositioning or wire adjustments. In addition, it may contribute to objective evaluation of overall treatment outcomes. With a median processing time ranging from 4 to 10 s per panoramic image, the system offers a clinically meaningful time advantage over manual measurement methods.

## 5. Conclusions

This study demonstrated that the artificial intelligence–based system yielded highly accurate and clinically reliable measurements of mesiodistal root angulation on panoramic radiographs. These results were very similar to the findings of expert manual assessment. The results indicate that AI-based assessment of root angulation could function as a rapid, objective, and reproducible guidance tool in orthodontic practice. Implementing these systems into standard panoramic analysis may improve treatment outcome assessment and facilitate clinical decision-making related to root parallelism.

## Figures and Tables

**Table 1:** Frequency distribution of exclusion criteria.

Reason for Exclusion	Number of OPG Excluded
Primary or mixed dentition period	3.187
Patients with retained primary teeth	165
Presence of impacted teeth (except third molars)	539
patients with missing teeth (one or more)	2.674
Presence of severe deep caries to an extent that prevents determination of the tooth’s long axis	81
Any pathologic lesions that may affect the positions of the roots	253
Dental anomalies that might affect the number or morphology of the teeth.	181
syndromes that may affect the dentition	4
presence of any artifact	453

## Data Availability

The data that support the findings of this study are available on re-quest from the corresponding author. The data are not publicly available due to privacy or ethical restrictions.

## Abbreviations

The following abbreviations are used in this manuscript:

AI	Artificial Intelligence
ML	Machine Learning
DL	Deep Learning
ANN	Artificial Neural Network
CNN	Convolutional Neural Network
OPG	Orthopantomogram (Panoramic Radiograph)
CBCT	Cone-Beam Computed Tomography
ICC	Intraclass Correlation Coefficient
